# Supplementary figures and images for: Cannabinoid type 2 receptor inhibition enhances the antidepressant and proneurogenic effects of physical exercise after chronic stress
Source: Transl Psychiatry. 2024 Mar 30;14:170. doi: 10.1038/s41398-024-02877-0 (PMC10981758; doi:10.1038/s41398-024-02877-0)

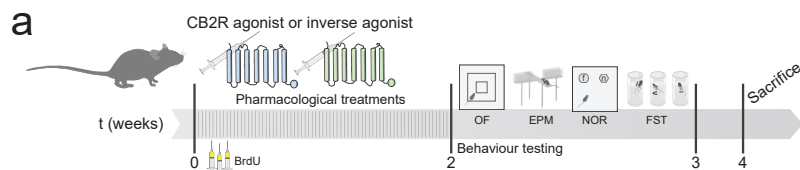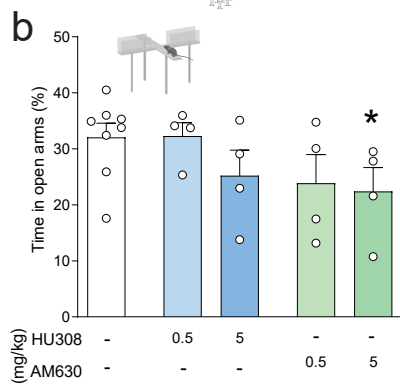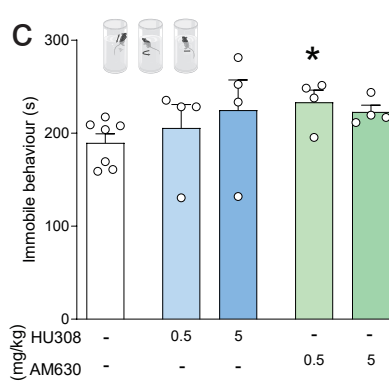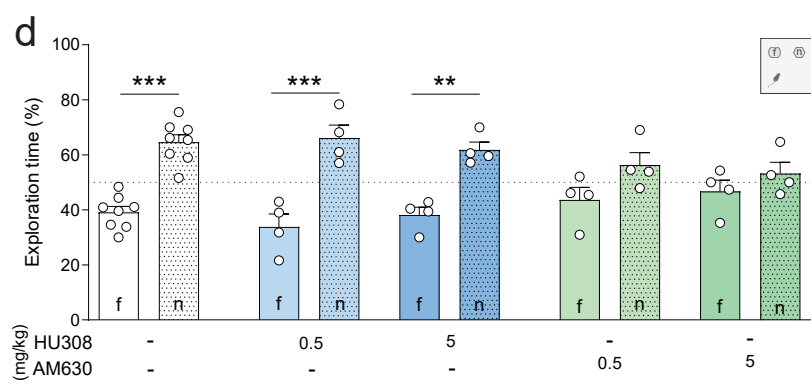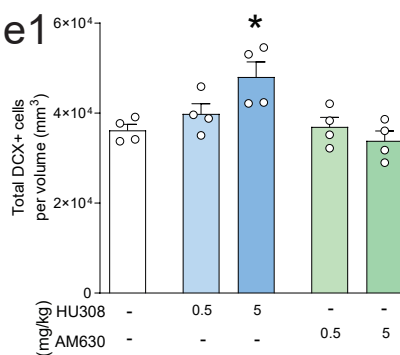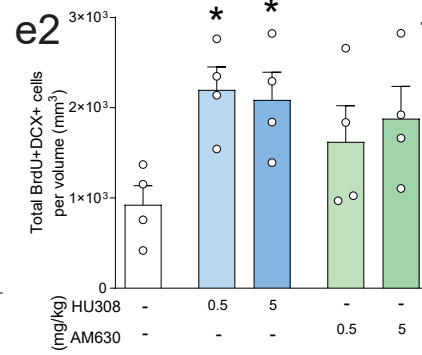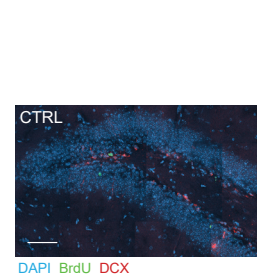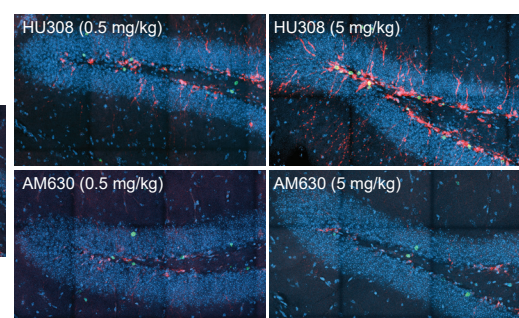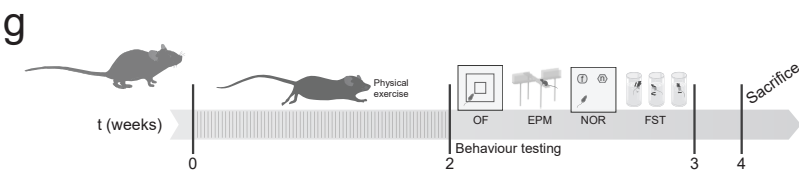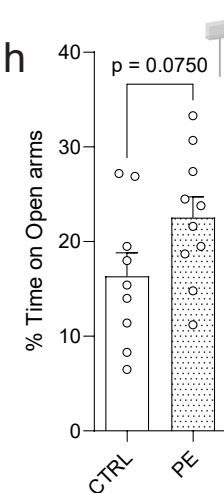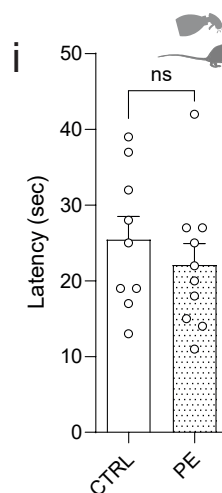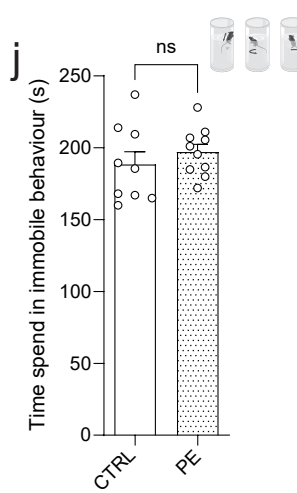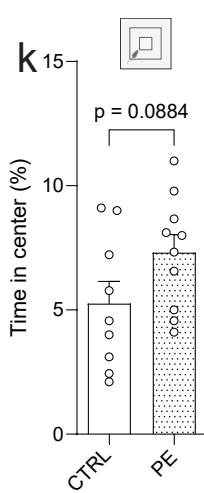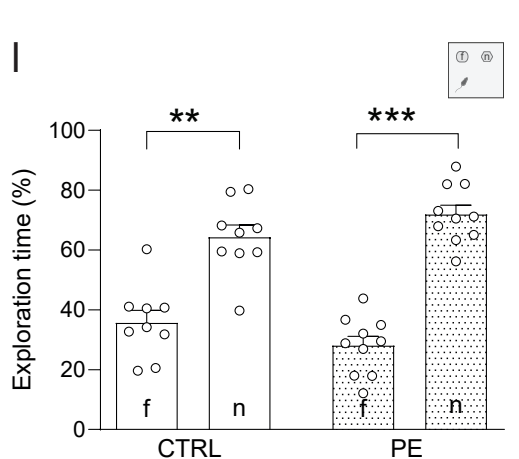

Supplement: Supplementary file 2 — Figure S1 [file 41398_2024_2877_MOESM2_ESM.pdf]

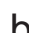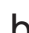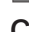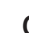

Supplement: Supplementary file 3 — Figure S2 [file 41398_2024_2877_MOESM3_ESM.pdf]

**a**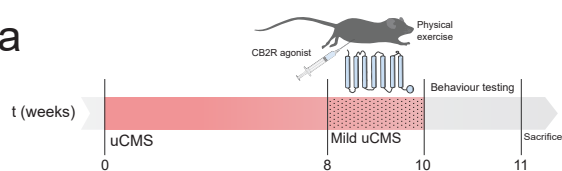**b**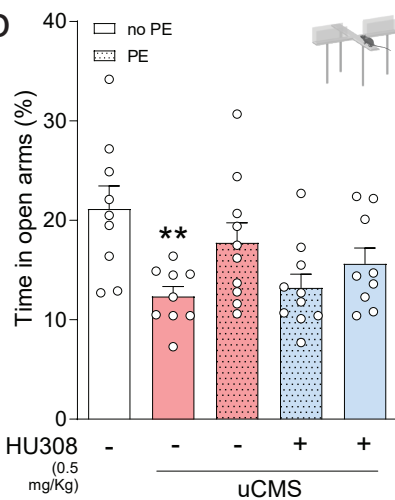**c**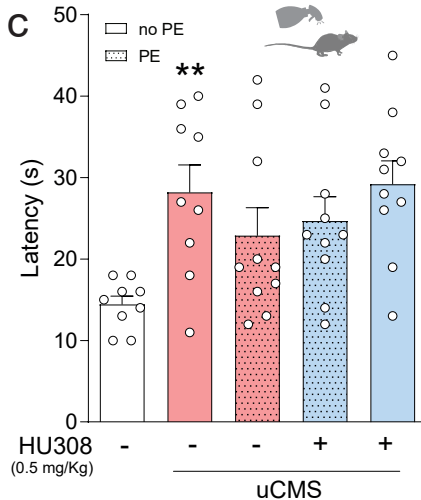**d**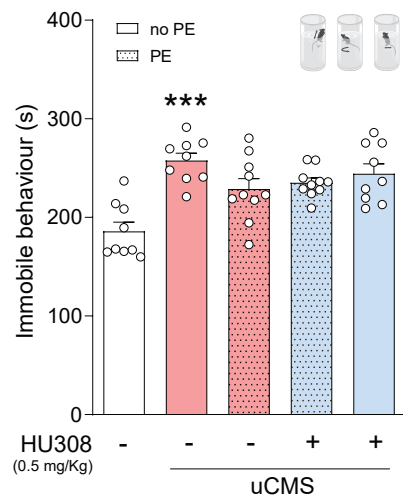**e**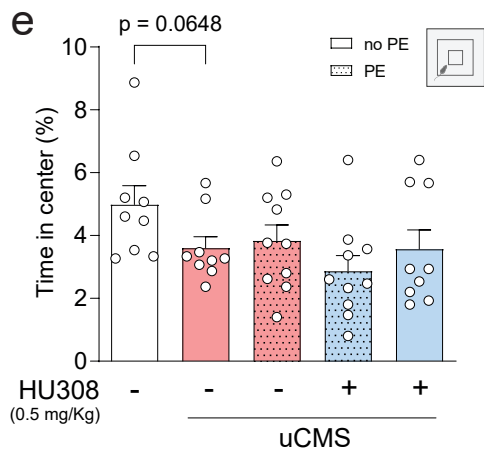**f**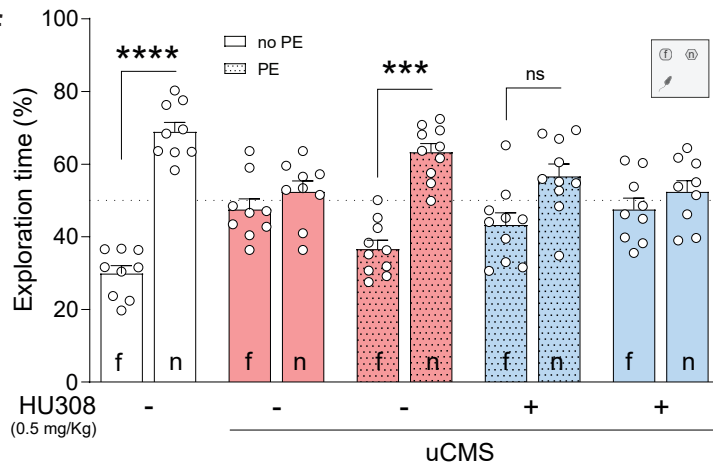

Supplement: Supplementary file 4 — Fig. S3 (related to Fig. 1) [file 41398_2024_2877_MOESM4_ESM.pdf]

## Granular expression

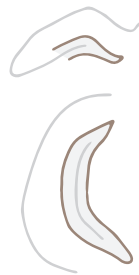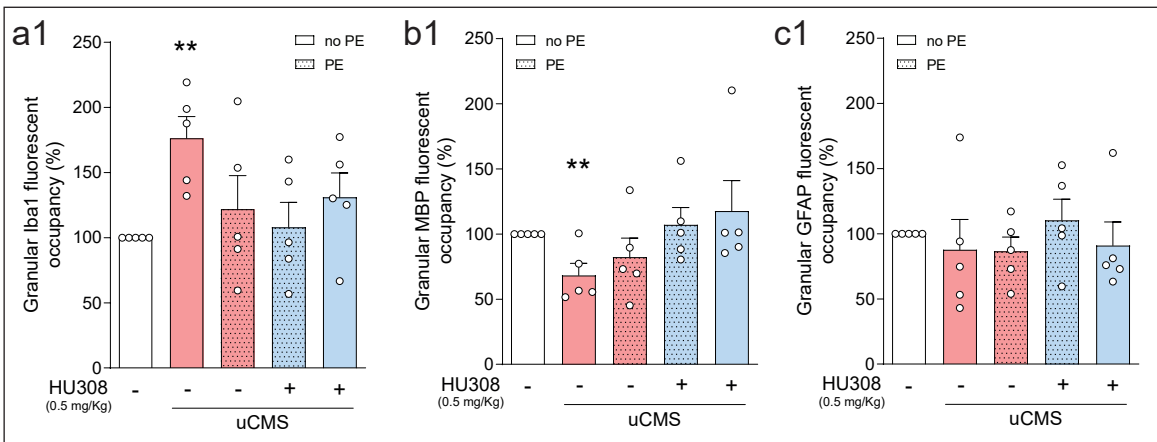

## Hilar expression

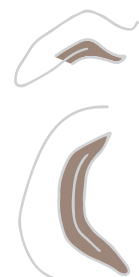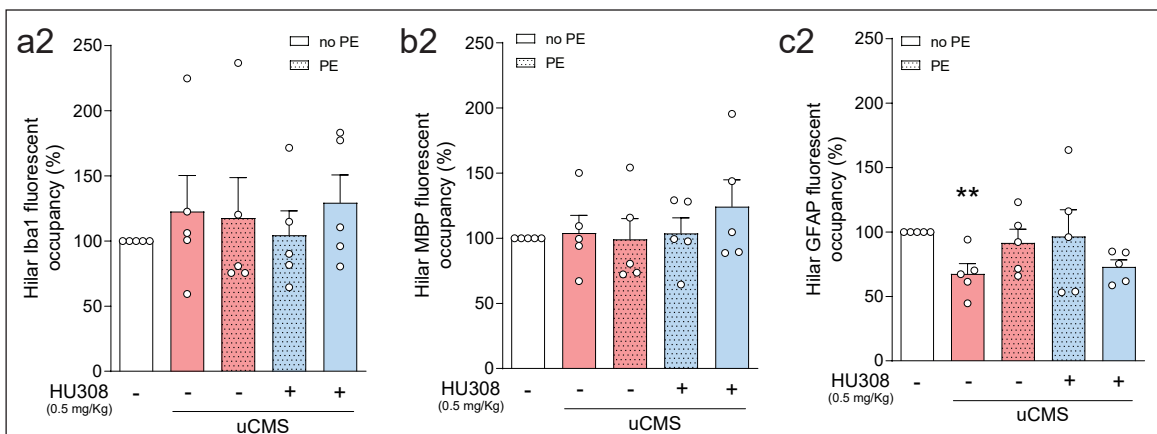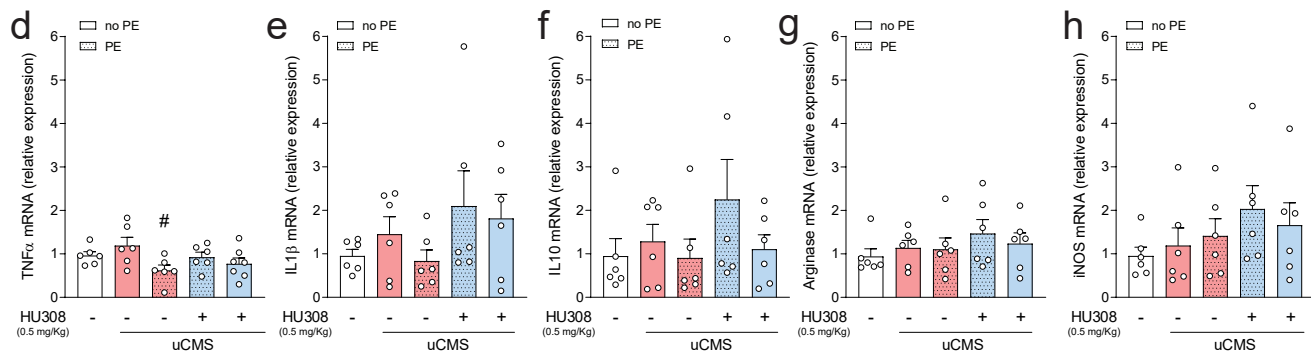

Supplement: Supplementary file 5 — Fig. S4 (related to Fig. 4) [file 41398_2024_2877_MOESM5_ESM.pdf]

a1

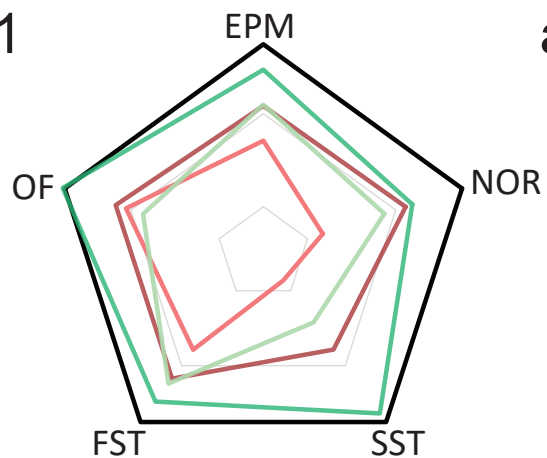

a2

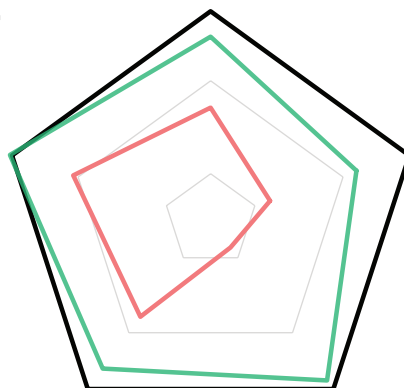

b1

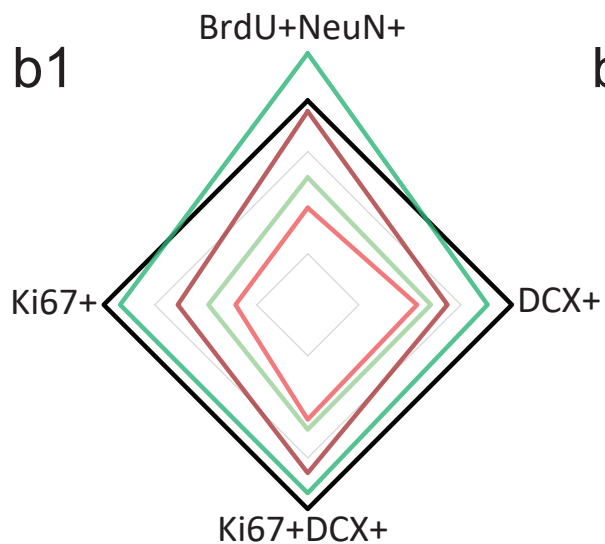

b2

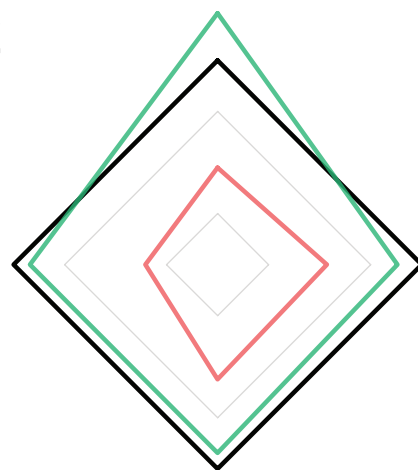

c1

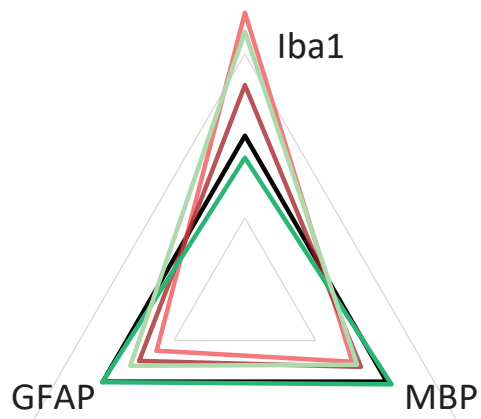

c2

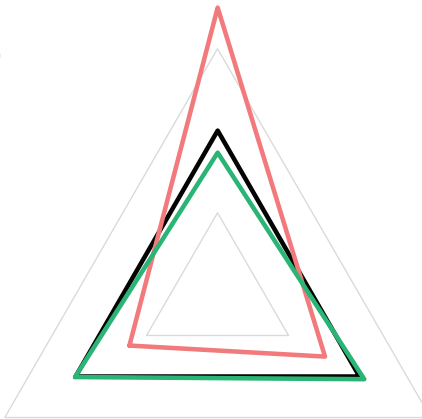

- CTRL
- uCMS
- uCMS+PE
- uCMS+AM
- uCMS+AM+PE

### Behaviour

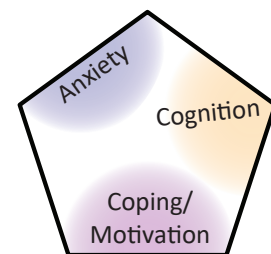

### Neurogenesis

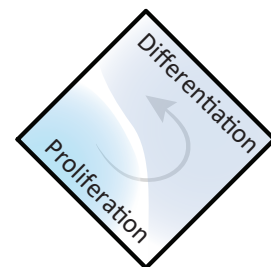

### Other cell types

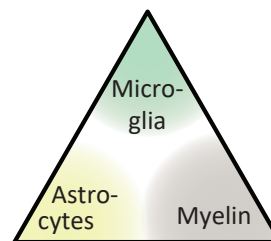

Supplement: Supplementary file 6 — Figure S5 [file 41398_2024_2877_MOESM6_ESM.pdf]
